# Responses of Maize Internode to Water Deficit Are Different at the Biochemical and Histological Levels

**DOI:** 10.3389/fpls.2021.628960

**Published:** 2021-02-26

**Authors:** Fadi El Hage, Laetitia Virlouvet, Paul-Louis Lopez-Marnet, Yves Griveau, Marie-Pierre Jacquemot, Sylvie Coursol, Valérie Méchin, Matthieu Reymond

**Affiliations:** ^1^Institut Jean-Pierre Bourgin, INRAE, AgroParisTech, Université Paris-Saclay, Versailles, France; ^2^Ecole Doctorale n° 567: Science du Végétal: Du gène à l’écosystème, Université Paris-Saclay, Orsay, France; ^3^Ecole Doctorale n° 581: ABIES, Paris, France

**Keywords:** maize internode, histological profile, cell wall biochemical composition, water deficit, quantitative trait locus

## Abstract

Maize feeding value is strongly linked to plant digestibility. Cell wall composition and structure can partly explain cell wall digestibility variations, and we recently showed that tissue lignification and lignin spatial distribution also contribute to cell wall digestibility variations. Although the genetic determinism of digestibility and cell wall composition has been studied for more than 20 years, little is available concerning that of tissue lignification. Moreover, maize yield is negatively impacted by water deficit, and we newly highlighted the impact of water deficit on cell wall digestibility and composition together with tissue lignification. Consequently, the aim of this study was to explore the genetic mechanisms of lignin distribution in link with cell wall composition and digestibility under contrasted water regimes. Maize internodes from a recombinant inbred line (RIL) population grown in field trials with contrasting irrigation scenarios were biochemically and histologically quantified. Results obtained showed that biochemical and histological traits have different response thresholds to water deficit. Histological profiles were therefore only modified under pronounced water deficit, while most of the biochemical traits responded whatever the strength of the water deficit. Three main clusters of quantitative trait locus (QTL) for histological traits were detected. Interestingly, overlap between the biochemical and histological clusters is rare, and one noted especially colocalizations between histological QTL/clusters and QTL for *p*-coumaric acid content. These findings reinforce the suspected role of tissue p-coumaroylation for both the agronomic properties of plants as well as their digestibility.

## Introduction

Maize is nowadays a staple food in the world (Shiferaw et al., 2011). It not only provides carbohydrates, proteins, lipids, and vitamins for billions of people but also serves as an important energy resource for animal feed and biorefinery processes (Virlouvet et al., 2019). Its energy value is first related to its cell wall digestibility.

Maize stem represents up to 46% of the plant dry weight (Hansey et al., 2010) and is made of distinct tissues, distributed in the rind and the pith sections, which do not have the same levels of lignification, *p*-coumaroylation, and feruloylation (Lopez et al., 1993; Chesson et al., 1997; El Hage et al., 2018; Santiago et al., 2018) nor the same efficiency of enzymatic digestibility (Lopez et al., 1993; Jung and Casler, 2006; El Hage et al., 2018). Therefore, spatial consideration of the maize stem will provide valuable knowledge to improve its utilization.

Recently, a non-lethal and cost-effective method has been proposed by puncture test (Seegmiller et al., 2020) to quantify rind thickness and stalk diameter on poison hemlock (*Conium maculatum*). Nevertheless, standardization of this method (for instance, puncturing probe geometry) is required to carry out meta-analysis of the results obtained by several groups (Cook et al., 2020). Other methods to quantify stem anatomy rely on the characterization of internode cross sections. An image processing software has been proposed to quantify rind thickness; pith area; vascular bundle number; and size in pith section of maize, sorghum, and other grass species (Heckwolf et al., 2015). Images are acquired on hand-cut cross section with a flatbed document scanner. The anatomical quantification rate required for genetic and physiological studies is therefore achieved. However, this method does not allow to demarcate the pith of the rind (Casto et al., 2018). In contrast, FASGA (abbreviation of Spanish names of fucsina, alcian blue, safranina, glicerina, and aqua) staining of internode cross sections (Tolivia and Tolivia, 1987) is a powerful approach that allows to distinguish highly lignified tissues and poorly lignified tissues. It was recently applied in sorghum (Perrier et al., 2017) and maize (El Hage et al., 2018; Vo et al., 2020) cross sections. Moreover, the advent of several plugins (Zhang et al., 2013; Legland et al., 2017) is allowing quantification of anatomical features from FASGA-stained cross sections, increasing the throughput of data analysis.

Variation of stem anatomical structure between genotypes has been revealed either by scoring visually the structure or by using the different quantification methods as described. For example, using a visual score, Casto et al. (2018) identified in different sorghum bi-parental populations a quantitative trait locus (QTL) on chromosome 6 that accounted for a large part of the variation in the amount of aerenchyma in these populations. Huang et al. (2016) similarly identified 16 QTL in a maize-teosinte population that accounted for 52% of the variation in the number of vascular bundle in the uppermost internode of that population. Using the protocol developed by Heckwolf et al. (2015); Mazaheri et al. (2019) also identified several candidate genes associated with maize stalk diameter, rind thickness, and vascular bundle density. However, to the best of our knowledge, no studies compare the position of QTL underlying biochemical trait variations to that of QTL underlying the variation of spatial distribution of lignified tissues.

In addition, severe episodes of drought are projected to become more frequent in the near future because of climate change (Harrison et al., 2014). We recently highlighted the impact of water deficit on cell wall digestibility and composition together with tissue lignification (El Hage et al., 2018; Virlouvet et al., 2019). Consequently, there is a need to elucidate the genetic determinism of lignin distribution in link with cell wall composition and digestibility for optimizing maize energetic value under water-limited conditions. To this end, the present study aimed at evaluating the biochemical and histological traits underlying the variation of cell wall digestibility in the maize stem and its plasticity in response to water deficit. A recombinant inbred line (RIL) population derived from the cross between F271 and Cm484 lines (Virlouvet et al., 2019) was evaluated during 2 years in the field under two contrasted irrigation scenarios. At final harvest, plant height, stem dry weight, biochemical composition, and tissue histology of the internode under the main ear were measured using high-throughput dedicated tools. A number of significant QTL for biochemical and histological traits were detected. Several colocalizations between histological QTL and QTL for PCAest were identified, supporting the major role of tissue *p*-coumaroylation for both the agronomic properties of plants as well as their digestibility.

## Materials and Methods

### Plant Materials and Field Experiments

A RIL population consisting of 267 lines was developed at INRAE by single seed descent (SSD) for six generations from a cross between maize inbred lines F271 and Cm484 as described (Virlouvet et al., 2019). All the RILs were planted in a randomized augmented block design in open field trials at Mauguio (France). Four replicates were carried out over 2 years (2014 and 2015) under irrigated (I) and non-irrigated (NI) conditions as described (Virlouvet et al., 2019). Briefly, under the I scenario, the water was supplied with a mobile ramp of sprinklers twice a week (30 mm of water supplied every time). Under the NI scenario, water was no more supplied when plants reached the five-leaf collar stage until 14 days after all the RILs flowered. Parental lines were replicated in each block of the design in 2014.I, 2014.NI, 2015.I, and 2015.NI. In 2014.I and 2014.NI, 201 plots were divided into eight blocks. In each block, parental lines F271 and Cm484 were cultivated in one plot each. RILs were cultivated on the other plots. At silage stage, among the 201 plots cultivated in 2014.I, stovers were harvested from 170 plots and internodes from 175 plots, due to heterogeneity of plants within a plot or due to low number of plants present in some plots. In 2014.NI, stovers were harvested from 170 plots and internodes from 174 plots. In 2015.I and NI, 189 plots were divided into eight blocks. In each block, parental lines F271 and Cm484 were cultivated in one plot each. At silage stage, stovers were harvested on 180 plots and internodes on 183 plots in 2015.I and stovers and internodes on 147 plots in 2015.NI. In each plot, 36 seeds from the same inbred line were sown and only the first internode below the main ear was harvested among the grown plants at silage stage. On one side, 10 internodes below the main ear of 10 plants were harvested and pooled, dried (in a forced-air oven at 55°C during 72 h), and grinded (with a hammer mill—1-mm grid) for NIRS acquisition. On the other side, two internodes below the main ear were harvested from two others plants and stored in 70% ethanol for histological quantifications. Hence, only internodes below the main ear were harvested for biochemical and histological quantification, avoiding differences due to different tissue sampling between biochemical predictions and histological quantifications. Each line was grown in a single 4.20-m row with 0.80 m between rows and a planting density of 80,000 plants ha^–1^.

### Biochemical Analyses and Establishment of NIRS Predictive Equations

First, the NIRS predictive equations for the internode previously described (El Hage et al., 2018) were enriched with the results from wet biochemistry analyses of 56 samples from the RIL population collected in Mauguio in 2014 and 2015. The 56 samples were selected to cover the range of spectral variation obtained on all the samples collected in 2014 and 2015 in irrigated and non-irrigated conditions. To do so, we performed a principal component analysis (PCA) carried out on NIRS spectrum acquired on all the harvested internode samples, and we selected 56 samples that were evenly distributed on the PCA axes. Biochemical quantifications performed on the calibration samples set were the following (Supplementary Table S1): (1) cell wall content (CWR) was obtained with a water/ethanol extraction (Soxhlet); (2) lignin content in the cell wall (KL.CWR) was estimated using the Klason method (Dence, 1992); (3) the monomeric structure and composition of lignin (units bO4.S, bO4.G, and bO4.H) were obtained by thioacydolysis (Lapierre et al., 1986); (4) PCAest, FAest, and FAeth were obtained by alkaline hydrolysis (Morrison et al., 1994; Culhaoglu et al., 2011); (5) glucose, xylose, and arabinose contents were quantified by acidic hydrolysis (Updegraff, 1969; Harholt et al., 2006), and cellulose content was estimated by glucose content, and hemicellulose content has been obtained by adding xylose and arabinose; and (6) acidic pretreatment of DM and CWR followed by an enzymatic hydrolysis (Onozuka cellulase R10) bring the *in vitro* DM degradability (IVDMD) and cell wall degradability (IVCWRD), respectively, as described (Virlouvet et al., 2019). Second, 683 DM internode samples were scanned through a near-infrared reflectance spectrometer (Antaris II, Thermo Fisher Scientific). The biochemical traits were then estimated using the updated NIRS predictive equations.

### Histological Analyses

Histological analyses have been carried out as described (Legland et al., 2017). Briefly, a 1-cm-long segment was sampled in the upper part of each internode 1.5 cm under the node. For each segment, one cross-section with a thickness of 150 μm was prepared. A total of 1,367 cross-sections were obtained using a sledge-microtome GSL1 (Gärtner et al., 2014) and stored in 70% ethanol. Sections were then stained for 24 h using a FASGA solution diluted in distilled water (1:8, *v*/*v*) and finally rinsed for 24 h with distilled water while stirring continuously. After two other successive washings during 5 min in MQ water, all the cross-sections were mounted in distilled water on slides and an image of each cross-section was acquired using a slide scanner piloted by the Metafer Scanning and Imaging Platform (MetaSystems GmbH, Altlussheim, Germany). Finally, each image was numerically analyzed on the ImageJ software to segment and quantify histological traits (Legland et al., 2017). The following nine histological traits were quantified:

- Stem_area, which is the area in square centimeter of the cross-section;
- Bundle_fraction, which is the percentage of the cross-section area occupied by the bundles;
- Bundle_number, which is the number of bundles in the pith of the cross-section;
- Lignified_fraction, which is the percentage of the cross-section area occupied by parenchyma stained by the safranin;
- Non-lignified_fraction, which is the percentage of the cross-section area occupied by parenchyma stained by the alcian blue;
- Rind_fraction, which is the percentage of the cross-section area occupied by the rind;

From all these traits quantified by the plugin, we calculated composite variables to synthetize the following information:

- Blue_parenchyma, which refers to the amount of blue intensity in the blue parenchyma;
- Red_parenchyma, which refers to the amount of red intensity in the red parenchyma;
- Red_rind, which refers to the amount of red intensity in the rind.

### Statistical Analyses

All statistical analyses were performed using R software (R Core Team, 2014). To eliminate the block effect, single-plot values were corrected by a subtraction of the best linear unbiased prediction (BLUP) value of the block effect for each line, using the following mixed linear model (1):

(1) $$Y_{\text{ijkl}}=\mu +g_{\text{i}}\times \left(1-t_{\text{i}}\right)+C_{\text{i}}\times y_{\text{i}}+e_{\text{k}}+B_{\text{jkl}}+g_{\text{i}}+y_{\text{i}}+g_{\text{i}}+e_{\text{k}}+y_{\text{i}}+e_{\text{k}}+E_{\text{ijkl}}$$

where *Y*_*ijkl*_ is the phenotypic value of the ith recombinant inbred line in the jth year, in the kth irrigation scenario and located in the lth block. μ is the intercept. Effect of line i is considered as fixed and noted *C*_*i*_ if it was one of the two parental lines used as control and as random and noted *g*_*i*_ if i was a RIL. The parameter *t*_*i*_ was set to one for control lines or set to zero otherwise. The *B*_*jkl*_ block was considered as random effects, as well as the interaction between the *g*_*i*_ genetic and the *y*_*j*_ year or the *e*_*k*_ irrigation scenario effects. The interaction between the *e*_*k*_ irrigation scenario and the *y*_*j*_ year effects was considered as fixed effect. By using the parental replicates in each block, the parameter *t*_*i*_ was used to distinguish the RILs from the parental lines. On the corrected data set, another analysis of variance (ANOVA) was performed with the following linear model for each trait:

*Y*_*ij*_ = μ + *A*_*i*_ + *T*_*j*_ + *A*_*i*_ × *T*_*j*_ + *R*_*ij*_

where *Y*_*ij*_ is the value of the year i under the condition j; μ = overall mean; *A*_*i*_ = the main effect of the year i; *T*_*j*_ = the main effect of the condition j; *A*_*i*_ × *T*_*j*_ = the interaction effect between the year i and the condition j; and *R*_*ij*_ = the random residual term. For each trait, heritabilities have been calculated over the years 2014 and 2015 either in I scenario or in NI scenario as the proportion of phenotypic variance explained by genotypic variance.

### Principal Component Analysis

Principal component analysis (PCA) was carried out with the complete data set through all the years and treatments using FactoMineR, factoextra, ggplot2, and ggthemes.

### QTL Detection

Genetic map of the population has been described previously (Virlouvet et al., 2019). Briefly, genomic DNA from 261 RIL was used for genotyping using the genotyping-by-sequencing (GBS) approach (Elshire et al., 2011). Initially, 955,720 markers were identified well-distributed on maize chromosomes 1–10. Among those, we selected 2,806 polymorphic markers between parental lines with less than 15% missing data among the RILs. These markers were then used to construct the linkage map using R scripts interacting with the CarthaGene software (de Givry et al., 2005), as described in Ganal et al. (2011). In total, 1,000 markers were retained. The total length of the framework map was 2,355 cM with an average spacing of around 2.4 cM. By looking *a posteriori* at singletons in the dataset, we verified that the increase of the genetic map length when saturating the scaffold with additional markers was not attributable to genotyping errors but more likely to the rather high level of missing data, which introduces a bias in the imputation procedure (EM algorithm) of CarthaGene as a result of genetic interference. QTL detection was performed using the multi-QTL mapping (MQM) method in the R/qtl package (Broman and Sen, 2009; Arends et al., 2010). The function mqmscan was used, setting the maximum cofactor marker number (number of individual minus 12, depending on the year and the condition), with a further processing by backward elimination. To quantify the explained variance, QTL above a LOD score at 3 (corresponding to approximately the 5% significance threshold using 1,000 permutations over all the quantified traits) were selected. The QTL detection by the MQM method was performed on the data corrected for the block effect per year and per irrigation scenario data. QTL confidence intervals were calculated as the genetic distance where LOD-1 from the QTL peak was reached.

## Results

### Internodes of F271 Are Less Digestible, Have More Lignified Tissues in the Rind and the Pith, and Are More Sensitive to the Irrigation Scenario Than Those of Cm484

Agronomic traits were first evaluated for the two parental inbred lines F271 and Cm484. Under the I condition, plant height and biomass yield were higher in F271 than in Cm484 in both 2014 and 2015 (Supplementary Table S2). Under the NI scenario, these two agronomical traits decreased as expected, but less strongly in 2015 than 2014 (Supplementary Table S2). Furthermore, their decrease was more pronounced in F271 (reduction of 59.5 and 39% of biomass yield in 2014 and 2015, respectively, in the NI vs. I scenario; reduction of 32.7 and 9.3% of plant height in 2014 and 2015, respectively, in the NI vs. I scenario) than in Cm484 (reduction of 44.2 and 7.8% of biomass yield in 2014 and 2015, respectively, in the NI vs. I scenario; reduction of 25.9 and −0.6% of plant height in 2014 and 2015, respectively, in the NI vs. I scenario).

Quantification of cell wall biochemical traits in the internodes showed that IVDMD and IVCWRD were lower in F271 than in Cm484 whatever the year and the irrigation scenario (Supplementary Table S2). F271 internode contained also more lignin in the cell wall (KL.CWR) than Cm484 whatever the year and the irrigation scenario (Supplementary Table S2). However, for both years, the lignin content was more lowered in F271 than in Cm484 in response to the irrigation scenario. The same trend was observed for the PCAest content (Supplementary Table S2). Reduction of cellulose content in the cell wall was also higher in internodes of F271 than in those of Cm484 in both years studied. In contrast, hemicellulose content in the cell wall of F271 internode was similar to that of Cm484 internode and was not impacted by the different irrigation scenarios. Lignin structure (βO4 yield) was also similar for both parental lines whatever the year and the irrigation scenario.

Histological analyses showed that the cross section area of Cm484 was bigger than that of F271 in 2014 whatever the irrigation scenario (Supplementary Table S2). However, in 2015, F271 and Cm484 showed similar cross section area whatever the irrigation scenario. Additionally, F271 showed a greater rind section area than Cm484 whatever the year and the irrigation (Supplementary Table S2). The lignified fraction in the pith was also more important for F271 than for Cm484 whatever the year and the irrigation scenario, suggesting that the irrigation scenario did not impact the lignified fraction in the pith. The main difference between the two parental lines was that Cm484 showed mostly twice the amount of non-lignified fraction in the pith than F271 whatever the year and the irrigation scenario (Supplementary Table S2). In line with this observation, the Blue_parenchyma (i.e., the amount of blue intensity in the blue parenchyma) was always higher in Cm484 than in F271 (Supplementary Table S2). It is also noticeable than the Blue_parenchyma increased in F271 internode while it decreased in Cm484 internode in response to the NI scenario in 2015 (Supplementary Table S2). Bundle numbers were similar between the two parental lines whatever the year and the irrigation scenario (Supplementary Table S2). However, due to the decrease of the cross section area in response to the NI scenario, the bundle density increased in the two parental lines in response to the irrigation scenario, with the exception of Cm484 in 2015 (Supplementary Table S2).

### Different Responses of Biochemical and Histological Traits to the Irrigation Scenario Among the F271 × Cm484 Progeny

**FIGURE 1:**
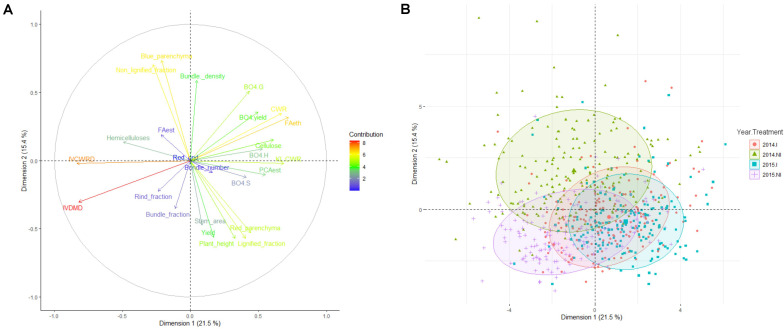
PCA analysis including histological, biochemical, and agronomical traits quantified. **(A)** Representation of the contribution of each trait in dimension 1 (21% of the global variance) vs. dimension 2 (16.3% of the global variance). **(B)** Projection of each recombinant inbred line for each year and each irrigation scenario in dimension 1 and 2 area of the PCA analysis. Circles represent the area occupied by 70% of the RIL for each year and treatment combination.

The agronomic and cell wall-related traits were then evaluated in the F271 × Cm484 progeny, and PCA analysis was carried out for investigating these traits over the years under both irrigation scenarios (Figure 1). The first axis of the PCA explained 21.5% of the variation present in the data set. The traits that contributed the most were IVCWRD and IVDMD on one side and FAeth, KL.CWR, CWR, cellulose, and PCAest on the other side (Figure 1A and Supplementary Table S3). Hence, this first ACP axis reflects the digestibility and the biochemical composition of the internode biomass. Internodes harvested under the I scenario in both 2014 and 2015 were located on the right side of this axis, whereas internodes harvested under the NI scenario in both 2014 and 2015 were located on the left side of this first PCA axis (Figure 1B). This reflects the fact that the digestibility and the biochemical composition of the internodes were modified by the NI scenario whatever the year. Under the NI scenario in both 2014 and 2015, IVCWRD increased while the lignin and PCAest contents in the cell wall decreased. Moreover, among the RIL population, the increase of IVCWRD in response to the NI scenario was similar in average for both years (Supplementary Table S2). The hemicellulose content in the cell wall increased slightly when internodes of the RILs were cultivated under the NI scenario in both 2014 and 2015, paralleled by a slight decrease of the cellulose content in 2014 and 2015.

The second axis of the PCA depicted 15.4% of the observed variability among the data set. The traits that contributed the most to this axis were the histological (Blue_parenchyma, Non-_lignified_fraction, and Bundle_density on one side and Lignified_fraction, Red_parenchyma, and Stem_area on the other side) and agronomical traits (Plant_height and yield) (Figure 1A and Supplementary Table S3). Internodes harvested when plant were cultivated under the I scenario in both 2014 and 2015 were located on the same position on this axis, while it was not the case for the internodes harvested under the NI scenario. Internodes harvested in 2015 were close to those harvested under the I scenario (Figure 1B). In contrast, internodes harvested under the NI scenario in 2014 were more distant than the others. This reflects the fact that the NI scenario in 2014 had a stronger impact on the histological and agronomical traits than the NI scenario in 2015 (Supplementary Table S2).

In accordance to the PCA results, comparing the correlations between traits within each field trials, the correlations obtained in 2014.NI differed from those obtained in 2015.NI (Supplementary Table S4). For instance, Blue_parenchyma is more strongly and significantly correlated to Red_rind and Red_parenchyma in 2014.NI than in the other field trials (*r* = −0.26^∗∗∗^ and *r* = −0.70^∗∗∗^, respectively, Supplementary Table S4). Bundle_density is significantly correlated to Non-_lignified_fraction and to IVCWRD only in 2014.NI on one side (*r* = 0.21^∗∗^ and *r* = −0.21^∗^, respectively, Supplementary Table S4) and it is also much less correlated to Bundle_number only in 2014.NI (*r* = 0.20^∗^; Supplementary Table S4). Correlation between lignified fraction and Rind_fraction is also weaker in 2014.NI than in the others field trials (*r* = -0.16^∗^; Supplementary Table S4). Blue_parenchyma, Non-_lignified_fraction, and Bundle_density are the traits that contributed the most to the second axis of the PCA. This reflects the fact that 2014.NI is located apart from the other field trials on the PCA and that histology of internodes responded more in 2014 to the NI scenario.

Taken together, the results suggest that the NI scenario led to internode modifications at the biochemical level of the same magnitude in 2014 and in 2015. However, changes in internodes at the histological levels were more pronounced in the NI scenario of 2014 than that of 2015.

### QTL Mapping for Histological, Biochemical, and Agronomical Traits Under the Different Irrigation Scenarios

**FIGURE 2:**
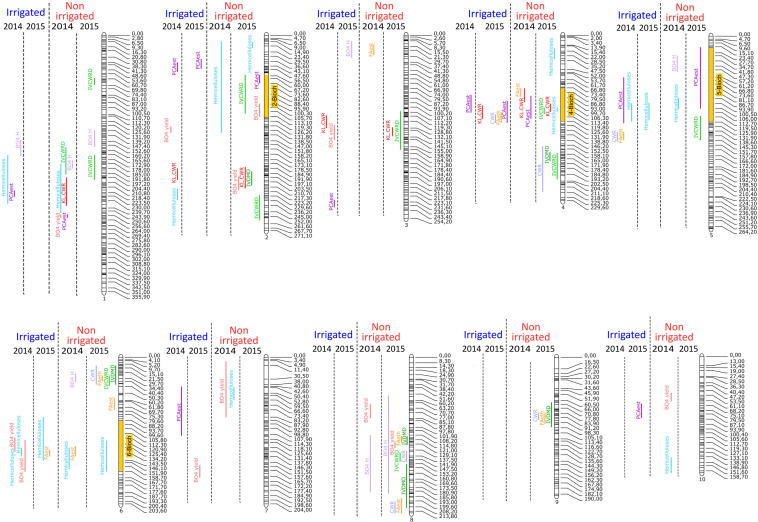
Mapping of QTL involved in the variations of biochemical traits on the 10 chromosomes of maize genome for the four conditions: 2014 irrigated, 2015 irrigated, 2014 non-irrigated, and 2015 non-irrigated.

**FIGURE 3:**
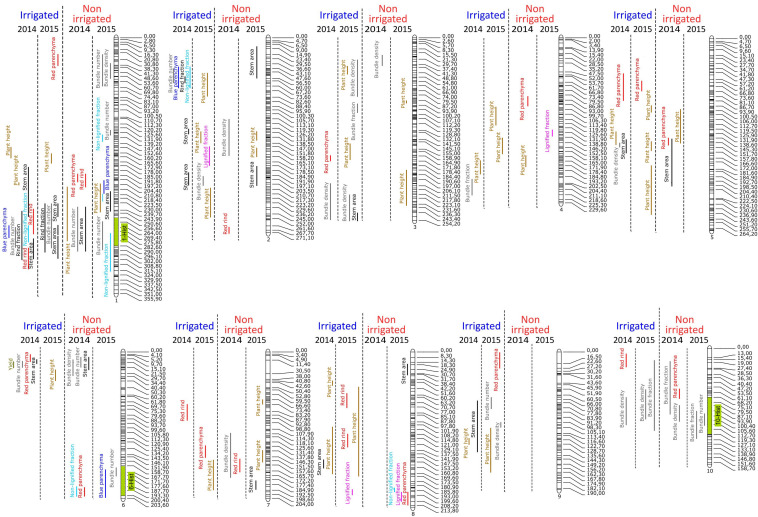
Mapping of QTL involved in the variations of histological and agronomical traits on the 10 chromosomes of maize for the four conditions: 2014 irrigated, 2015 irrigated, 2014 non-irrigated, and 2015 non-irrigated.

Consequently, QTL detection was performed for agronomical, histological, and biochemical traits for each year and each irrigation scenario. For all year and scenario combinations (2014.I, 2014.NI, 2015.I, and 2015.NI), a total of 219 QTL involved in the variations of the quantified agronomical, biochemical, and histological traits were mapped (Figures 2,3 and Supplementary Table S5).

For the biochemical traits, a total of 94 QTL were detected over the 2 years and the two irrigation scenario tested. Several colocalizations occurred at several loci between QTL for digestibility and/or QTL for composition/structure of the cell wall (Figure 2 and Supplementary Table S5). Interestingly, among the 94 QTL detected, 30 mapped to four clusters. At cluster 2-Bioch (between 50 and 105 cM on the chromosome 2), one QTL for IVCWRD (2015.NI) colocalized with QTL for PCAest (2015.NI), hemicelluloses (2014.NI), and βO4 yield (2015.NI). At cluster 4-Bioch (between 73 and 117 cM on chromosome 4), a QTL for IVCWRD (2015.NI) colocalized with QTL for CWR (2015.I), KL.CWR (2015.I, 2014.NI and 2015.NI), PCAest (2014.I, 2015.I and 2014.NI), FAest (2015.I), hemicelluloses (2015.NI), and FAest (2014.NI). At cluster 5-Bioch (between 10 and 114 cM on chromosome 5), QTL for hemicelluloses (2014.I, 2015.I and 2014.NI) colocalized with QTL for PCAest (2014.I and 2015.NI), IVCWRD (2015.NI), and βO4 H (2014.NI). At cluster 6-Bioch (between 82 and 155 cM on chromosome 6), QTL for hemicelluloses detected in every condition colocalized with QTL for FAest (2015.I and 2014.NI) and βO4 yield (2014.I). If globally no locus seemed to be associated to the irrigation scenario, it is noteworthy that some QTL appeared exclusively under the NI scenario. This was the case at the top of the chromosome 6 (between 7 and 70 cM) and on chromosome 8 in which several biochemical QTL were detected only in 2014.NI and 2015.NI conditions (Figure 2 and Supplementary Table S5).

For the histological traits, a total of 90 QTL were detected (Figure 3 and Supplementary Table S5). The percentage of variation explained by each histological QTL was rather low and only six QTL explained more than 10% of the observed variation of the histological trait. Interestingly, among the 90 histological QTL identified, 25 mapped to three clusters (Figure 3). The 1-Hist cluster on chromosome 1 (between 250 and 290 cM) included 14 histological QTL detected whatever the year and the irrigation scenario. The 10-Hist cluster on chromosome 10 (between 80 and 105 cM) included seven QTL involved only in the variation of bundle-related traits (Bundle_number, Bundle_density, and Bundle_fraction) whatever the year and the irrigation scenario. The 6-Hist cluster on the bottom of chromosome 6 (between 158 and 200 cM) included only four QTL for histological traits under the NI scenario in both 2014 and 2015. It did not overlap with the 6-Bioch cluster. Additionally, four QTL involved only in histological traits in the I scenario in both 2014 and 2015 were mapped on chromosome 9.

Finally, several colocalizations were observed between histological and biochemical traits. The 1-Hist cluster included two biochemical QTL (one QTL for PCAest and one QTL for βO4 yield) detected in 2014 in the NI scenario and one QTL for Plant_height detected as well in 2014 in the NI scenario. It is worth noting that QTL for PCAest content often colocalized with QTL involved in the variation of histological traits (Figure 3). Thus, at the top of chromosome 2, three QTL for PCAest (2014.I, 2014.NI, and 2015.NI) colocalized with three histological QTL (Rind_fraction, Blue_parenchyma, and Bundle_number) detected in 2014 under the I scenario. Similarly, at the bottom of chromosome 3, QTL for PCAest colocalized with QTL for Bundle_density detected in 2014 in the I scenario. On the chromosome 5, QTL for PCAest (2014.I and 2015.NI) also colocalized with QTL for Red_Parenchyma (2014.I and 2015.I). On chromosome 7, QTL for PCAest (2014.I) again colocalized with QTL for Red_rind detected under the same condition. Finally, on the chromosome 10, QTL for PCAest (2015.I) colocalized with the 10-Hist cluster.

## Discussion

### Combining NIRS Predictions and Histological Profiling of Maize Internode to Decipher Biochemical and Histological Variations

Cm484 was previously reported to have a blue FASGA-stained parenchyma in the pith while F271 presented a much more reddish FASGA-stained parenchyma (Méchin et al., 2000; Méchin et al., 2005; Barrière et al., 2009). Based on these findings, a RIL population was developed from a cross between these two inbred lines. El Hage et al. (2018) recently characterized a set of maize inbred lines, including F271 and Cm484, and confirmed their biochemical differences. Surprisingly, Cm484 did not show a so intense blue FASGA-stained parenchyma anymore, making its histological profile much closer from that of F271. In the present study, Cm484 again did not exhibit an intense blue FASGA parenchyma. Nevertheless, we were still able to highlight histological differences between the two parental lines, and we showed that indeed the Non-lignified_fraction of Cm484 was more present widespread in the parenchyma than that of F271. More importantly, transgression was observed for all agronomical-, biochemical-, and histological-quantified traits in the RILs, which allowed us to detect QTL for most of these traits. These quantifications were possible thanks to two high-throughput phenotyping tools. First, NIRS equations were established to accurately predict traits related to cell wall composition and structure as well as *in vitro* digestibilities (IVDMD and IVCWRD) in the internodes of the RIL population. Second, we used a high-throughput image analysis (Legland et al., 2017) to quantify histological traits from FASGA-stained maize internode cross sections. These two tools have enabled us to accurately characterize biochemical and histological traits in the mapping population. This is an important step compared to previous internode studies that only highlighted the link between the distribution of the lignification and cell wall degradability in maize (Jung and Casler, 2006; Boon et al., 2008; El Hage et al., 2018), sugarcane (Dos Santos et al., 2015), and sorghum (Perrier et al., 2017) using a small set of lines. A number of QTL for vascular bundle system or stem aerenchymas have been mapped in plants of agronomical interest such as in tomato (Coaker et al., 2002), wheat (Sang et al., 2010), rice (Sasahara et al., 1999; Zhang et al., 2002; Cui et al., 2003; Bai et al., 2012), and in sorghum (Casto et al., 2018). In maize, the genetic determinism of histological traits has also recently been investigated (Huang et al., 2016; Mazaheri et al., 2019; Zhang et al., 2020). Here, the use of FASGA-stained cross section allowed us to delimit the rind and the pith fractions and to differentiate level of lignification in the different fractions of maize internode.

Moreover, until now, no link was made between the variation of histological pattern and the variation of cell wall digestibility and composition.

### Responses to Water Deficit Are Different When Considering Biochemical or Histological Traits

All lines were cultivated in field trials, where irrigation was either provided (I scenario) or stopped (NI scenario) in 2014 and 2015. The natural precipitation did not occur in the same way during the maize growing season in the 2 years: Indeed, a storm occurred on June 10th 2015, providing up to 80 mm of water under both I and NI conditions. The amount of water supply during this storm represented more water than all the rainfalls cumulated during the entire maize growing season in 2014. Thus, all the plants under the NI condition in 2015 benefited from a great water supply at the start of the onset of water stress. Plant height and biomass yield were less impacted in 2015 than in 2014 by the NI scenario, reflecting the differences between 2014 and 2015. Moreover, note that in the physiological development of the plant, the 10th of June coincides with the period of the development of the internode carrying the main ear. Consequently, the NI condition of 2014 and 2015 could not be considered similar at the internode level. This gives us the opportunity to compare the impact of two very different stress intensities. The significance of the year and treatment × year effects for most of the traits reflects this difference between 2014 and 2015.

We showed that most of the biochemical traits were impacted by the NI scenario in both years. These findings have already been reported in maize (El Hage et al., 2018; Virlouvet et al., 2019), sorghum (Luquet et al., 2019), and miscanthus (Van Der Weijde et al., 2017). Nevertheless, what is striking in the present analysis is that even if rainwater was supplied in large quantities from the onset of the stress period and of the development of the internode below the ear in 2015, biochemistry traits (such as PCAest and lignin contents) and *in vitro* digestibilities of the internodes were impacted similarly in 2014 and 2015, while the histological traits were not. Histological and agronomical traits contributed the most to the emergence of the second PCA axis, and only data from 2014 in the NI scenario were projected apart from the others on this second PCA axis, reflecting the fact that the NI scenario impacted histological pattern only in 2014 but not in 2015. According to El Hage et al. (2018), the histological responses observed in 2014 resulted in smaller internodes, with a smaller bundle area but greater density, and a proportionately larger non-lignified parenchyma area.

### Distinct QTL Mapped for Biochemical and Histological Traits

Heritabilities on the un-replicated RIL field trials have been estimated either over the I scenario and the NI scenario carried out in 2014 and 2015 for biochemical and histological traits (Supplementary Table S2). Overall, for most of the traits, heritability in I scenario are higher than the ones in NI scenario. This corroborates with the fact that 2014_I and 2015_I trials are more similar than 2014_NI and 2015_NI trials (Figure 1). Moreover, heritability for most of the biochemical traits remained moderated (from 12.4 to 54.5%, except for FAest that reached only 7.6%). This maintenance of the value for heritability for most of the chemical traits goes along with the fact that biochemical traits responded similarly each year. Under I scenario, heritability of histological trait is in accordance with what is reported (rind thickness *h*^2^ = 58% in Mazaheri et al., 2019; bundle-related traits *h*^2^ from 12.8 to 83.6% in Zhang et al., 2020). However, histological traits were only impacted by NI scenario in 2014 but not in 2015 (see Figure 1 and section “Discussion”). This leads to a pronounced reduction of estimated heritability for histological traits in NI scenarios, and it forced us to continue the identification of QTL trial by trial. Hence, the low heritability for some traits reported in Supplementary Table S2 do not reflect the fact that we identify QTL for these traits under each field trial.

So far, numerous QTL studies have been reported on traits related to agronomic performance and cell wall composition and digestibility. The present study allows to map over 200 QTL for agronomical, biochemical, and histological traits under two contrasting water regimes in the field. It is noteworthy that the QTL for plant height identified on chromosomes 5 (2014.I and 2014.NI), 8 (2014.I and 2015.NI), and 9 (2014.I and 2015.I) colocalized with QTL for the same trait previously identified in a set of 800 maize inbred lines (Mazaheri et al., 2019). Furthermore, the QTL of stem area mapped on chromosomes 1 (2014.I), 2 (2014.I and 2015.NI), 5 (2014.NI), and 7 (2015.NI) colocalized with loci involved in stalk diameters measured on the third internode previously identified (Mazaheri et al., 2019). QTL involved in bundle numbers (chromosomes 1, 6, and 10 under 2015.NI) also colocalized with those previously identified in the uppermost internode of an 866 maize-teosinte BC2S3 RILs (Huang et al., 2016). In contrast, the QTL for Rind_fraction on chromosome 5 did not colocalize with the only association Mazaheri et al. (2019) reported for rind thickness. Additionally, the 1-Hist cluster colocalized with a locus involved in the variation of PCAest previously identified in the pith of the second internode below the main cob in a panel of 282 divers maize lines (López-Malvar et al., 2019). Moreover, at this position, a QTL for PCAest content has been also reported by Santiago et al. (2016). These authors also detected QTL for PCAest at the top of chromosome 2 and on chromosome 4, positions that colocalize with QTL for PCAest mapped in our study. The 1-Hist cluster did not colocalize with any association found by Zhang et al. (2020) using a panel of 482 lines and computed tomography (Du et al., 2016). Nevertheless, the QTL for Bundle_number on chromosome 1 at 61.4 Mb colocalized with some of their associations. These differences may be due to the fact that the studied internodes and the methods used differ.

At the biochemical level, four clusters of QTL were detected. It is noteworthy that only one them, the cluster 4-Bioch, encompassed QTL involved in the variation of both lignin content and *p*-hydroxycinnamic acid contents in the cell wall. Furthermore, three biochemical clusters (4-Bioch, 5-Bioch, and 6-Bioch) can be considered as constitutive since QTL for both years and irrigation scenarios have been detected at these loci. Interestingly, the clusters 4-Bioch and 6-Bioch colocalized with clusters of constitutive QTL detected on chromosome 4 and 6, respectively, at the whole-plant level (Virlouvet et al., 2019). In addition, QTL for biochemical traits identified only in the NI scenario (for instance on the bottom of chromosome 8) colocalized with responsive QTL mapped at the whole-plant level (Virlouvet et al., 2019). This suggests genetic links for cell wall biochemistry and its response to water deficit between the internode and whole-plant levels. Indeed, correlations for biochemical traits at the internode level and at the stover level (Virlouvet et al., 2019) have been calculated (Supplementary Table S6). For each field trial (2014.I, 2014.NI, 2015.I, and 2015.NI), correlations were significant for CWR, KL.CWR, FAest, PCAest, and for the *in vitro* digestibilities. This is in agreement with the correlation between digestibility of the stem and that of the stover previously reporter (Hansey et al., 2010; El Hage, 2018).

At the histological level, three clusters of QTL were detected. Among them, two (1-Hist and 10-Hist) included QTL detected whatever the year and the irrigation scenario. They are therefore constitutively involved in the variation of the histological profile of maize internode. On the other hand, the 6-Hist cluster was mapped only in the NI condition in both 2014 and 2015. It is worth noting that no overlap between histological and biochemical clusters occurred in this population. However, we frequently noticed colocalizations between QTL for PCAest content and QTL for histological variations mainly of rind and lignified parenchyma area and/or intensity. This is in accordance with the fact that these tissues are reported to be highly *p*-coumaroylated tissues (Lopez et al., 1993; Chesson et al., 1997; Barros-Rios et al., 2012).

## Conclusion

We validated the feasibility of high-throughput tools developed to quantify biochemical and histological traits for QTL detection or for selection screening. Our work led to the identification of constitutive QTL, as well as QTL with an effect only in the NI condition, suggesting that the genetic determinism involved in cell wall development at the biochemical and histological levels are distinct from those involved under the NI scenario. Overall, our data highlighted the potential role of *p*-coumaroylation of maize stem in refining plant agronomic properties and digestibility.

## Data Availability Statement

The original contributions presented in the study are included in the article/Supplementary Material, further inquiries can be directed to the corresponding author/s.

## Author Contributions

MR, VM, and SC designed the study and supervised data collection. FEH, M-PJ, SC, VM, and MR collected the samples. FEH prepared the internode cross sections and quantified histological traits. YG, M-PJ, and VM carried out the biochemical traits quantification. YG established the NIRS predictive equations. FEH, P-LL-M, LV, and MR conducted the genetic study. FEH, P-LL-M, SC, VM, and MR wrote the manuscript. All authors contributed to the article and approved the submitted version.

## Conflict of Interest

The authors declare that the research was conducted in the absence of any commercial or financial relationships that could be construed as a potential conflict of interest.

## Supplementary Material

Supplementary Table 1 Characteristics of NIRS predictive equations developed for cell wall traits in maize internodes harvested at silage stage.

Supplementary Table 2 Variations and heritabilities of each histological, biochemical, and agronomical trait observed for the parental lines and for the RILs cultivated in 2014 and 2015 in both I and NI scenarios. Different letters reported for the parental values indicated significant differences from a Tukey test.

Supplementary Table 3 Cos2 and Pearson correlation of all the traits for the two first axes of the PCA.

Supplementary Table 4 Pearson correlation table of all the biochemical and histological traits for the RILs in each field trial: 2014.I, 2014.NI, 2015.I, and 2015.NI.

Supplementary Table 5 List of QTL detected for all the histological, biochemical, and agronomical traits. When the effect sign is positive, Cm484 alleles are responsible of the increase of the trait; when the effect sign is negative, the alleles from F271 are increasing the trait value.

Supplementary Table 6 Correlations between the biochemical traits quantified at the internode and at the stover levels (Virlouvet et al., 2019).
